# The dynamic network characteristics of physical, psychological, and cognitive symptoms of people with HIV based on cross-lagged network analysis in China

**DOI:** 10.1371/journal.pone.0340077

**Published:** 2026-03-20

**Authors:** Meilian Xie, Xiaoyu Liu, Zhiyun Zhang, Yanping Yu, Li Zhang, Jieli Zhang, Dongxia Wu

**Affiliations:** 1 Nursing Management Department, Beijing Ditan Hospital Capital Medical University, Beijing, China; 2 Nursing Management Department, Capital Center for Children’s Health, Capital Medical University, Capital Institute of Pediatrics, Beijing, China,; 3 School of statistics and data science, Capital University of Economics and Business, Beijing, China; 4 Beijing Home of Red Ribbon, Beijing Ditan Hospital Capital Medical University, Beijing, China; 5 Infection and Immunization Clinic, Beijing Ditan Hospital Capital Medical University, Beijing, China; 6 Department of Infectious Disease Medicine, Fifth Medical Center of Chinese PLA General Hospital, Beijing, China; 7 Department of Infection and Immunology, Beijing Youan Hospital Capital Medical University, Beijing, China; Para Federal University, BRAZIL

## Abstract

**Introduction:**

This study aims to construct a dynamic network by collecting longitudinal data, which will assist medical professionals in identifying bridging symptoms associated with disease progression and causal mechanisms.

**Methods:**

A longitudinal observational study was conducted from March to June 2024, recruiting an initial cohort of people with HIV (PWH) from three designated AIDS medical institutions in Beijing, China. Symptom data were collected at two time points, three months apart, using the Chinese version of the Self-Report Symptom Scale (SRSS) alongside a demographic questionnaire. The data were analyzed using cross-lagged panel network analysis to explore symptom dynamics over time.

**Results:**

A total of 791 PWH were recruited and 706 participants (89.25%) continued to the 3-month follow-up, with a mean age of 38.37 ± 10.03 years. There were no significant differences in demographic variables between those who missed and those who completed the study, except for clinical indices such as CD4 + cell count, ART duration, and comorbidity. Psychological symptoms, such as feeling helpless, feeling uneasy, and feeling fearful, also appear frequently. The most significant association identified was between becoming confusing and having difficulty in reasoning (COGS5 → COGS4, OR = 1.20). Notable bridging symptoms across two distinct domains included becoming confusing, leading to hardly focusing on anything (COGS5 → PSYS2, OR = 1.12), feeling hopeless, leading to rash (PSYS8 → PHYS8, OR = 1.11), and worry overwhelming leading to sleep disturbance (PSYS3 → PHYS6, OR = 1.11), among others.

**Conclusions:**

There is an urgent need to enhance the assessment and early monitoring of cognitive status for PWH.

## Introduction

As of now, China has reported 740,787 people with HIV (PWH). While the overall number of infected individuals has shown a downward trend compared to previous years, the rising incidence of new infections continues to be a significant cause for concern [1].The implementation of China’s policy of free antiretroviral therapy (HAART) for PWH in 2004 [2] substantially lengthened the life expectancy of PWH, approaching that of the general population [3]. But living longer does not necessarily mean living healthily. UNAIDS emphasized that improving the quality of life for PWH was a key indicator of treatment success in the Global AIDS Strategy 2021–2026 [4].

The combination of treatment-related adverse reactions and disease-associated discomforts and comorbidities often worsens the perceptions and severity of symptoms in PWH. A survey by Fudan University’s nursing team involving 1,116 PWH from five cities in southern China (2017–2019) found an average of 9 reported symptoms per patient [5]. Similarly, a survey by Beijing Ditan Hospital’s nursing team, including 711 PWH from nine regions across China (2020–2021), reported an average of 5 symptoms [6]. The presence of multiple symptoms significantly impacts patients’ quality of life and clinical outcomes [7], highlighting the importance of understanding the correlations between these symptoms for better symptom management.

With the advancement of information technology, symptom networks have emerged as a novel technology that elucidates the complex relationships between symptoms. It has been initially applied and explored in the fields of psychopathology, chronic disease symptom management, and long-term follow-up of tumors [8–10]. It is widely accepted that one or more symptoms with high centrality, referred to as bridging symptoms, exist within the symptom network. These bridging symptoms can initiate negative cycles by activating other symptoms, ultimately leading to the progressive deterioration of the patient’s physical and mental functions [11,12]. Compared to previous studies, this network analysis method provides a more intuitive demonstration of the significance of the associations among multiple symptoms throughout the disease process. Identifying bridging symptoms within the network can aid in pinpointing intervention targets, thereby offering a precise and efficient approach to clinical practice.

The application of symptom networks in HIV is still in its early stages. Researchers from Fudan University and the nursing team at Beijing Ditan Hospital have used cross-sectional data to create a static symptom network for PWH, analyzing the relationships between symptom clusters [5]. They identified bridging symptoms influenced by depression and anxiety [13], offering new directions for precise care in China. However, real-world symptoms are complex and dynamic. Unlike static networks, dynamic networks better reflect disease trends and evolving symptom relationships [14–16]. Specifically, cross-lagged networks capture the temporal correlations between symptoms over time, providing insights into relationships and identifying clinical targets. Despite this, research on HIV symptom correlations and relationship mechanisms from a dynamic network perspective remains limited. Therefore, this study aims to build a dynamic network using longitudinal data to help identify bridging symptoms linked to disease progression.

## Methods

### Study design and participants

This study is longitudinal research with a three-month interval between two data collection waves (SMAIDS PROGRAMME). The 3-month interval was selected to capture clinically meaningful symptom fluctuations in chronic, virologically suppressed HIV while minimizing recall bias and attrition. The data was collected from 22/03/2024 to 24/6/2024 at three medical institutions designated for AIDS diagnosis and treatment in Beijing, China. The first measurement was conducted from March to June 2024 (Time 1), and the second follow-up survey occurred three months later in late June 2024 (Time 2). All study participants were reimbursed for their time (RMB 35 per visit). Recruitment of participants took place through the display of posters in outpatient clinics. We enrolled PWH who were 18 years or older, were undergoing outpatient treatment at three specified medical centers, had been on ART for a minimum of one month, showed at least one symptom in the two weeks leading up to the survey, and possessed a smart device they could use with the necessary support. Those unable to finish the questionnaire due to severe health issues or diagnosed with neurocognitive or visual impairments were excluded from our study. All participants gave their written informed consent online. The study protocol is available online at ClinicalTrials.gov (ChiCTR2400080468) on Jan 30, 2024.

### Measurements

#### Demographic and clinical variables.

Participants provided demographic details, such as age, gender, race, and several measures of socioeconomic status, including education level, marital status, employment status, and household income. Furthermore, clinical information was gathered via the electronic medical record system, encompassing the duration of HIV, disease stage, CD4 cell count, length of ART, and any comorbidities.

#### Self-Report Symptom Scale.

The Chinese version of the Self-Report Symptom Scale (SRSS) [17] was used to assess symptom experiences (frequency, severity, and distress) in PWH. The scale was expanded from 27 to 28 items by adding one item from Tsai’s adaptation of the Sign and Symptom Checklist [18]. Additionally, the study incorporated 8 new symptom items from the PROMIS framework to replace 5 psychological items, bringing the total to 32 items. The scale demonstrated a high reliability with a Cronbach’s α coefficient of 0.94 (with a range between 0.92 to 0.95). It evaluates physical, cognitive, and psychological symptoms over the past two weeks, with frequency and severity assessed using a four-point Likert scale, and distress measured on a five-point scale. The total experience score for each symptom is the sum of the three dimensions.

### Procedure and data collection

Medical staff working in outpatient clinics at the three medical institutions served as field investigators, having received uniform training before the formal study beginning. The field investigators were responsible for monitoring the entire process and establishing the research files for the participants. After recruiting into this study, participants completed the initial investigation on-site. All data collected were managed by two researchers who strictly adhered to confidentiality protocols. The on-site survey was conducted face-to-face, with investigators guiding participants in completing the online questionnaire via (https://www.wjx.cn/). Participants took approximately 10–15 minutes to complete all questions. All questions in the questionnaire did not involve the participants’ personal privacy. Each participant was assigned a number as a proxy for their name from the day they joined the study. Subsequent follow-up surveys utilized this designated number as the unique identification method.

### Ethics approval and consent to participate

The Ethics Review Board of Beijing Ditan Hospital, Capital Medical University, approved this study, and the reference number is NO.DTEC-KY2023-049-02. We confirm that all methods were performed in accordance with the Declaration of Helsinki, and written informed consent was obtained from all participants.

### Data analysis

The cross-lagged panel network analysis (CLPN) was calculated using logistic regression models to assess autoregressive effects (where a symptom at T1 predicts its own value at T2, accounting for other symptoms and covariates) and cross-lagged effects (where a symptom at T1 has an association with a different symptom at T2, after controlling for other symptoms and covariates). In our study, since most individual edges or network density were not interpretable, regression coefficients were regularized using LASSO with 10-fold cross-validation to select the tuning parameter, shrinking smaller coefficients to zero. This approach suggests that the estimated CLPN likely reflects the true network structure but may also include some false positives and overestimations in density. For the CLPN analysis, the total symptom experience scores (sum of frequency, severity, and distress dimensions) were treated as continuous variables. We made the decision to include only age and sex as covariates in the CLPN without introducing over-adjustment bias for the symptom network structure.

The coefficient of odds ratios (ORs) enhances interpretability. An edge weight that exceeds 1 suggests a positive association, while an edge weight falling below 1 signifies a negative association, and an edge weight equal to 1 implies no association. The glmnet package in R and the qgraph package were utilized to compute regularized regressions or visualize network [19,20]. Expected influence (EI) serves as a metric that encapsulates the number, intensity, and nature of the connections between a specific symptom and all other symptoms in the network. The CLPN is a directed network modeled from longitudinal data. We differentiated the directionality by individually computing the cross-lagged out-EI (defined as the aggregation of values from outgoing connections associated with a symptom), in-EI (defined as the aggregation of values from incoming connections linked to a symptom), and the bridge EI aiming to pinpoint bridge symptoms [21].

The precision and consistency of edge weights were assessed using two bootstrapping techniques available in the R package bootnet [22] Initially, the accuracy of edge weights was determined by calculating 95% confidence intervals (CIs) around each edge weight through nonparametric bootstrapping, which involved 1,000 iterations. Subsequently, case-drop bootstrapping was utilized to compute correlation stability (CS) coefficients to evaluate the stability of the rank-order of centrality indices. Additionally, tests for differences in centrality were performed to ascertain if the variations among node centralities were statistically significant.

## Results

### Characteristics of the sample

**Fig 1:**
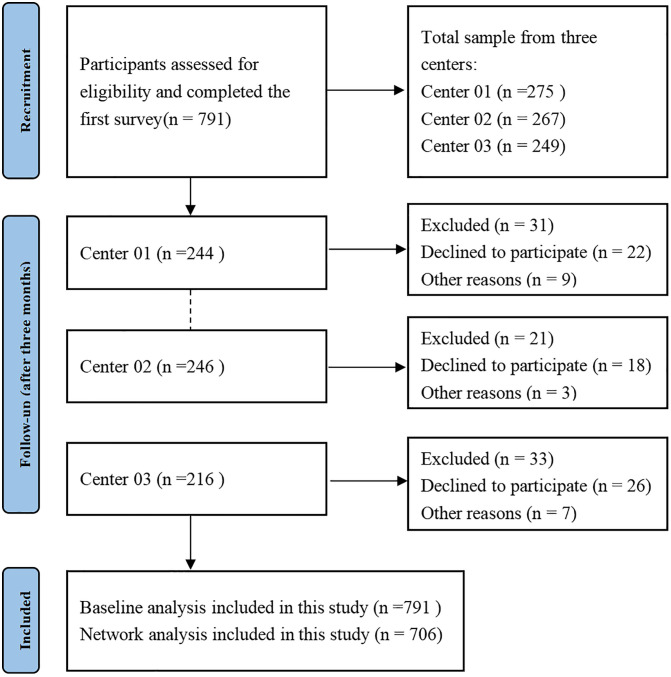
Flow chart.

In this study, 791 PWH from three hospitals were initially assessed for eligibility and completed the first survey. After three months, 85 PWH were lost to follow-up for various reasons, while 706 were included in the final network analysis (see **Fig 1**). The study sample (n = 791) in **Table 1** had a mean age of 38.37 years (SD = 10.03), with no significant age difference between those with missing data and those who completed the study. Most PWH were male (90.23%) and Han ethnicity (92.21%). The largest education level was Junior college or Undergraduate (52.12%), and 61.90% were single. Over half (54.96%) were employed, while 44.62% lived independently. A significant portion (53.96%) had an income above 6001 RMB, and 87.54% resided in urban areas. HIV duration varied, with 37.96% infected for 6 ~ 10 years, and 55.95% had CD4 counts above 500 (*P* ＜ 0.01). Most participants (39.24%) had received ART for 6 ~ 10 years (*P* = 0.02), and 83.99% of completers had no comorbidities (*P* = 0.03).

**Table 1:** Demographic characteristics of this sample.

Variables	Total (n = 791)	Missing (n = 85)	Completion (n = 706)	statistic	*P*-value
**Age, Mean ± SD**	38.37 ± 10.03	36.31 ± 8.57	38.61 ± 10.16	4.04	0.05
**Gender, n (%)**				0.06	0.81
Male	713 (90.14)	76 (89.41)	637 (90.23)		
Female	78 (9.86)	9 (10.59)	69 (9.77)		
**Race, n (%)**				Fisher	0.67
Han	731 (92.41)	80 (94.12)	651 (92.21)		
Minority	60（7.59）	5（5.88）	55（7.79）		
**Education, n (%)**				3.01	0.39
Middle school or below	129 (16.31)	14 (16.47)	115 (16.29)		
High school or equivalent	189 (23.89)	17 (20.00)	172 (24.36)		
Junior college or Undergraduate	419 (52.97)	51 (60.00)	368 (52.12)		
Postgraduate	54 (6.83)	3 (3.53)	51 (7.22)		
**Marital status, n (%)**				0.20	0.91
Single	491 (62.07)	54 (63.53)	437 (61.90)		
Married or cohabiting	195 (24.65)	21 (24.71)	174 (24.65)		
Others	105(10.49)	10 (11.76)	95 (13.46)		
**Employment, n (%)**				Fisher	0.77
Student	20 (2.53)	2 (2.35)	18 (2.55)		
Employed	439 (55.50)	51 (60)	388 (54.96)		
Unemployed	51 (6.45)	4 (4.71)	47 (6.66)		
Freelancer	241 (30.47)	26 (30.59)	215 (30.45)		
Retired	40 (5.06)	2 (2.35)	38 (5.38)		
**Type of residential population, n (%)**				1.13	0.57
Living alone	354 (44.75)	39 (45.88)	315 (44.62)		
Living with family	303 (38.31)	35 (41.18)	268 (37.96)		
Living with friends/others	134 (16.94)	11 (12.94)	123 (17.42)		
**Household income, n (%)**				2.02	0.57
<3000 RMB	104 (13.15)	8 (9.41)	96 (13.6)		
3000 ~ 6000 RMB	260 (32.87)	31 (36.47)	229 (32.44)		
6001 ~ 10000 RMB	237 (29.96)	23 (27.06)	214 (30.31)		
>6000 RMB	190 (24.02)	23 (27.06)	167 (23.65)		
**Region, n (%)**				1.80	0.18
Urban	688 (86.98)	70 (82.35)	618 (87.54)		
Rural	103 (13.02)	15 (17.65)	88 (12.46)		
**HIV duration, n (%)**				7.48	0.06
<1 years	78 (9.86)	13 (15.29)	65 (9.21)		
1 ~ 5 years	265 (33.50)	35 (41.18)	230 (32.58)		
6 ~ 10 years	293 (37.04)	25 (29.41)	268 (37.96)		
>10 years	155 (19.60)	12 (14.12)	143 (20.25)		
**Diagnostic method, n (%)**				1.83	0.61
Hospitalization examination	159 (20.10)	13 (15.29)	146 (20.68)		
Medical checkup	265 (33.50)	28 (32.94)	237 (33.57)		
Self-check	315 (39.82)	37 (43.53)	278 (39.38)		
Others	52(6.58)	7 (8.24)	45 (6.37)		
**Staging of disease, n (%)**				Fisher	0.15
Acute stage	19 (2.40)	2 (2.35)	17 (2.41)		
Asymptomatic stage	527 (66.62)	49 (57.65)	478 (67.71)		
AIDS stage	245 (30.97)	34 (40)	211 (29.89)		
**CD4 cell count, n (%)**				13.92	＜0.01*
<200	126 (15.93)	25 (29.41)	101 (14.30)		
200 ~ 499	235 (29.71)	25 (29.41)	210 (29.75)		
≥500	430 (54.36)	35 (41.18)	395 (55.95)		
**ART duration, n (%)**				9.436	0.02*
<1 years	75 (9.48)	13 (15.29)	62 (8.78)		
1 ~ 5 years	293 (37.04)	39 (45.88)	254 (35.98)		
6 ~ 10 years	299 (37.80)	22 (25.88)	277 (39.24)		
>10 years	124 (15.68)	11 (12.94)	113 (16.01)		
**Comorbidity, n (%)**				7.109	0.03*
0	654 (82.68)	61 (71.76)	593 (83.99)		
1	106 (13.40)	19 (22.35)	87 (12.32)		
2	31 (3.92)	5 (5.89)	26 (3.69)		

### Symptom and endorsement rates

**Fig 2:**
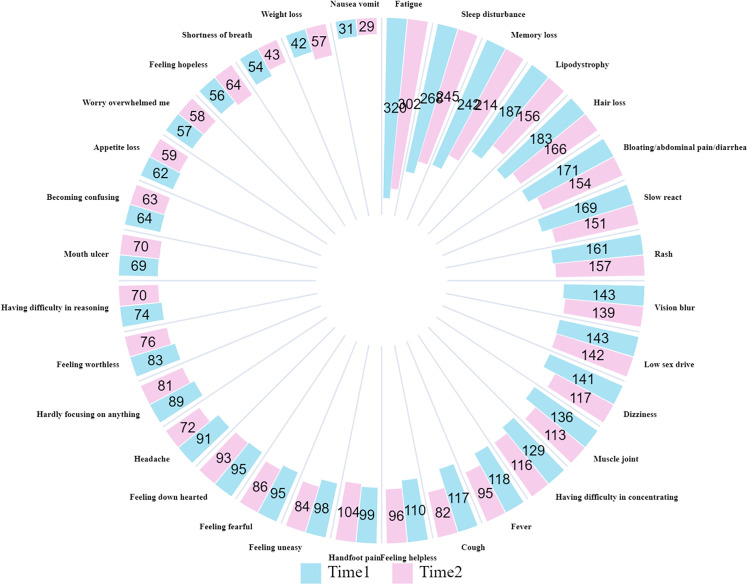
Number of occurrences of each symptom at different times.

**Fig 2** presents a circular bar chart comparing the frequency of various symptoms at two time points: Time 1 and Time 2. Fatigue and sleep disturbances remain the most common symptoms at both time intervals. Most symptoms exhibited a notable decline at Time 2, whereas weight loss and hand/foot pain showed a moderate increase during this period. Psychological symptoms like feeling helpless, feeling uneasy, and feeling fearful appear frequently. The chart highlights the persistence and fluctuation in symptom prevalence, underscoring the complexity of symptom progression over time.

### Network inference

**Fig 3:**
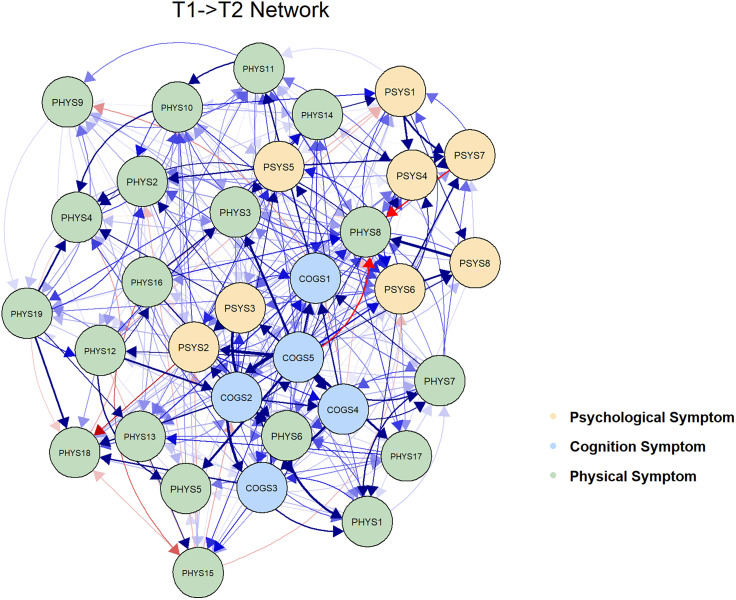
The cross-lagged panel networks for T1 → T2 with arrows representing dynamic relationships. Blue edges indicate positive relationships, while red edges indicate negative relationships. The thickness of the edges corresponds to the strength of the odds ratio, with thicker edges representing stronger relationships. To enhance visual interpretation, auto regressive edges, weaker edges (i.e., odds ratios below 1.35), and covariates have been excluded from the plot. PHYS1: Fatigue; PHYS2: Dizziness; PHYS3: Headache; PHYS4: Fever; PHYS5:Cough; PHYS6: Sleep disturbance; PHYS7:Vision blur; PHYS8: Rash; PHYS9: Mouth ulcer; PHYS10: Muscle/joint ache; PHYS11: Hand/foot pain; PHYS12: Appetite loss; PHYS13: Bloating/abdominal pain/diarrhea; PHYS14: Nausea/vomit; PHYS15: Lipodystrophy; PHYS16: Weight loss; PHYS17: Low sex drive; PHYS18: Hair loss; PHYS19:Shortness of breath; COGS1: Having difficulty in concentrating; COGS2: Slow react; COGS3: Memory loss; COGS4: Having difficulty in reasoning; COGS5: Becoming confusing; PSYS1: Feeling fearful; PSYS2: Hardly focusing on anything; PSYS3: Worry overwhelmed me; PSYS4: Feeling uneasy; PSYS5:Feeling worthless; PSYS6:Feeling helpless; PSYS7: Feeling downhearted; PSYS8: Feeling hopeless.

The CLPN model was used to estimate the T1 → T2 symptom network (see **Fig 3**), illustrating the complex interplay among psychological, cognitive, and physical symptoms across two time points. Strong autoregressive effects exist for most symptoms in the network. All autoregressive edges were present (see **S1 Fig**), and autoregressive edges (mean OR = 1.16) were slightly stronger than cross-lagged edges (mean OR = 1.01)（**Table 2**.

**Table 2:** Adjacency matrix of the T1 → T2 cross-lagged panel network.

Item	PHYS1	PHYS2	PHYS3	PHYS4	PHYS5	PHYS6	PHYS7	PHYS8	PHYS9	PHYS10	PHYS11	PHYS12	PHYS13	PHYS14	PHYS15	PHYS16	PHYS17	PHYS18	PHYS19	COGS1	COGS2	COGS3	COGS4	COGS5	PSYS1	PSYS2	PSYS3	PSYS4	PSYS5	PSYS6	PSYS8
**PHYS1**	1.15	1	1	1	1	1.03	1.01	1	1	1	1	1	1.01	1	1	1	1	1	1	1.01	1.01	1.03	1	1	1	1	1	1	1	1	1
**PHYS2**	1	1.14	1.02	1.04	1	1.02	1	1.01	1.01	1.01	1	1.01	1	1.01	1	1	1	1.02	1.01	1	1.02	1	1	1.01	1	1.01	1	1	1	1	1
**PHYS3**	1	1.02	1.12	1	1	1	1	1	1	1	1.02	1	1	1	1	1	1	1	1	1.03	1.02	1	1	1	1	1	1.01	1	1	1.03	1
**PHYS4**	1	1	1	1.13	1	1	1	1	1	1.03	1	1	1	1	1	1	1	0.99	1	1	1	1	1	1	1	1	1	1	1	1	1
**PHYS5**	1	1	1	1	1.15	1	1	1	1	1.01	1	1	1	1	1	1	1	1	1.01	1	1	1	1	1	1	1	1	1	1	1	1
**PHYS6**	1.09	1.02	1	1.01	1	1.19	1	1	1	1	1	1	1	1	1.01	1	1	1	1	1.02	1	1.03	1	1	1	1.02	1	1	1	1	1
**PHYS7**	1.05	1.02	1.02	1	1	1.03	1.24	1.02	1	1	1.02	1.01	1	1	1	1.01	1	1	1	1.04	1	1.02	1.02	1.01	1	1	1	1	1	1	1
**PHYS8**	1	1	1.01	1.04	1	1.02	1	1.25	1	1.01	1	1	1	1.01	1	1	1	1	1	1.01	1.02	1.01	1	1.01	1	1	1	1	1	1	1
**PHYS9**	1	1	1	1	1.01	1	1	1	1.27	1	1.02	1	1	1	1	1	1	1	1.01	1	1	1	1.01	1	1	1	1	1	1	1.01	1
**PHYS10**	1	1.03	1	1.05	1	1	1	1.03	1	1.14	1.02	1	1.02	1.01	1	1	1	1	1.01	1	1	1.01	1	1	1.03	1	1	1	1.01	1.03	1
**PHYS11**	1	1	1.01	1	1	1	1.02	1.02	1.02	1.05	1.19	1	1	1	1	1	1	1	1	1	1	1	1	1	1	1	1	1	1	1.03	1
**PHYS12**	1	1	1	1	1.06	1	1	1	1	1.02	1	1.14	1	1.03	0.99	1.05	1	1	1.02	1	1.07	1	1	1	0.99	1	1	1	1	1	1
**PHYS13**	1	1	1	1	1	1.02	1.01	1.03	1	1	1	1	1.17	1	1.04	1	1	1.02	1	1	1.02	1	1	1	1	1	1	1	1	1	1
**PHYS14**	1	1	1.06	1	1	1	1	0.99	1	1	1	1	1.02	1.06	1	1	1.03	1	1	1.03	1	1	1	1	1.05	1	1	1.04	1	1	1
**PHYS15**	1	0.99	1	1	1.01	1	1	1	1	1.01	1	1	1	1	1.22	1	1	0.99	1	1	0.99	1	1	1	1	0.99	1	1	1	0.99	1
**PHYS16**	1	1	1.05	1	1	1.06	1.01	1.04	1	1	1.02	1.01	1.01	1	0.98	1.21	1.02	1	1.01	1	1	1	1	1	0.99	1	1	1	1	1	1
**PHYS17**	1	1	1	1.01	1.02	1.02	1	1	1	1	1	1.01	1.03	1	1	1.02	1.21	1	1	1	1	1	1	1	1	1	1	1	1	1	1
**PHYS18**	1.01	1	1	0.99	1	1	1	1.01	1	1	1	1	1	1	1	1	1	1.23	1	1	1	1	1	1	1	1	1	1	1	1	1
**PHYS19**	1	1.03	1	1.07	1	1.01	1	1	1.02	1	1	1.04	1.05	1	1	1	1	1.07	1.17	1	1	1	1	1	1	1	1	1	1	1.02	1
**COGS1**	1	1	1.01	1	1	1	1	1.03	1	1.01	1.05	1	1.01	1	1.03	1	1	1.01	1	1.17	1	1	1	1	1	1.03	1	1	1	1	1
**COGS2**	1.02	1	1	1	1	1.05	1	1	1.01	1.01	1.01	1	1.02	1	1	1	1	1.05	1	1.03	1.21	1.11	1.06	1.04	1.02	1.05	1.03	1.02	1.04	1.04	1
**COGS3**	1.06	1.01	1.01	1.03	1	1.05	1.01	1.01	1.01	1.01	1	1	1	1	1.04	1	1.02	1.07	1	1	1.07	1.16	1	1	1.01	1	1	1.03	1	1.01	1
**COGS4**	1.02	1	1	1	1	1.01	1.04	0.99	0.98	1	1	1	1	1.02	1.01	1	1.01	1	1	1.06	1	1.1	1.05	1	1	1	1	1	1	1	1
**COGS5**	1.02	1	1.09	1	1.1	1	1.06	0.94	1	1	1.02	1.04	1	1.02	1	1.01	1.08	1.01	1.01	1.07	1.14	1	1.2	1.27	1.01	1.12	1.08	1.04	1.04	1.03	1.04
**PSYS1**	1	1	1	1	1	1	1	1	1	1	1	1	1	1	1	1	1	1	1	1	1	1	1	1	1.17	1	1.02	1.08	1	1.03	1.04
**PSYS2**	1	1	1	1	1	1	1	1	1	1.03	1	1	1	1	1	1	1	0.96	1	1.02	1	1	1.03	1	1	1.05	1	1.02	1.03	1.01	1
**PSYS3**	1.01	1.04	1	1.05	1	1.11	1	1	1	1	1	1	1.01	1	1	1	1	1	1	1	1	1	1	1	1	1.05	1.08	1.01	1	1	1.01
**PSYS4**	1	1.02	1	1	1	1	1	1.02	1	1	1	1	1	1	1	1	1	1.01	1	1	1	1	1	1	1	1	1	1.05	1	1.04	1
**PSYS5**	1	1.06	1	1	1	1	1	1.01	1	1	1.01	1.01	1.01	1	1	1	1	1	1.03	1	1	1	1	1.01	1	1	1.01	1	1.14	1.02	1
**PSYS6**	1.05	1	1	1	1	1	1.01	1.03	1	1	1	1	1.02	1	1.01	1	1.02	1	1	1	1	1.02	1	1	1.02	1.02	1.02	1.04	1.03	1.17	1.07
**PSYS7**	1	1	1	1	1	1	1	0.94	1	1	1	1	1	1	1	1	1	1	1	1	1	1	1	1	1.02	1	1	1.04	1	1	1
**PSYS8**	1.01	1	1	1.01	1	1	1	1.11	1	1	1.01	1	1.01	1	1	1	1	1	1	1	1	1	1	1	1	1	1.02	1	1	1	1.11

Independent variables (i.e., predictors) are in rows, and dependent variables are in columns. Autoregressive edges are presented along the diagonal.

PHYS1: Fatigue; PHYS2: Dizziness; PHYS3: Headache; PHYS4: Fever; PHYS5:Cough; PHYS6: Sleep disturbance; PHYS7:Vision blur; PHYS8: Rash; PHYS9: Mouth ulcer; PHYS10: Muscle/joint ache; PHYS11: Hand/foot pain; PHYS12: Appetite loss; PHYS13: Bloating/abdominal pain/diarrhea; PHYS14: Nausea/vomit; PHYS15: Lipodystrophy; PHYS16: Weight loss; PHYS17: Low sex drive; PHYS18: Hair loss; PHYS19:Shortness of breath; COGS1: Having difficulty in concentrating; COGS2: Slow react; COGS3: Memory loss; COGS4: Having difficulty in reasoning; COGS5: Becoming confusing; PSYS1: Feeling fearful; PSYS2: Hardly focusing on anything; PSYS3: Worry overwhelmed me; PSYS4: Feeling uneasy; PSYS5:Feeling worthless; PSYS6:Feeling helpless; PSYS7: Feeling downhearted; PSYS8: Feeling hopeless

The findings indicate that the T1 → T2 phase encompasses 336 cross-lagged connections, with 314 (93.45%) exhibiting an OR exceeding 1. The three most significant connections are: Becoming confusing→Having difficulty in reasoning (COGS5 → COGS4, OR=1.20), Becoming confusing→Slow react (COGS5 → COGS2, OR=1.14), and Slow react→Memory loss (COGS2 → COGS3, OR=1.11). The most prominent bridge symptoms across two distinct domains include Becoming confusing→Hardly focusing on anything (COGS5 → PSYS2, OR=1.12), Feeling hopeless→Rash (PSYS8 → PHYS8, OR=1.11), Worry overwhelmed me→Sleep disturbance (PSYS3 → PHYS6, OR=1.11), and so on.

**Fig 4:**
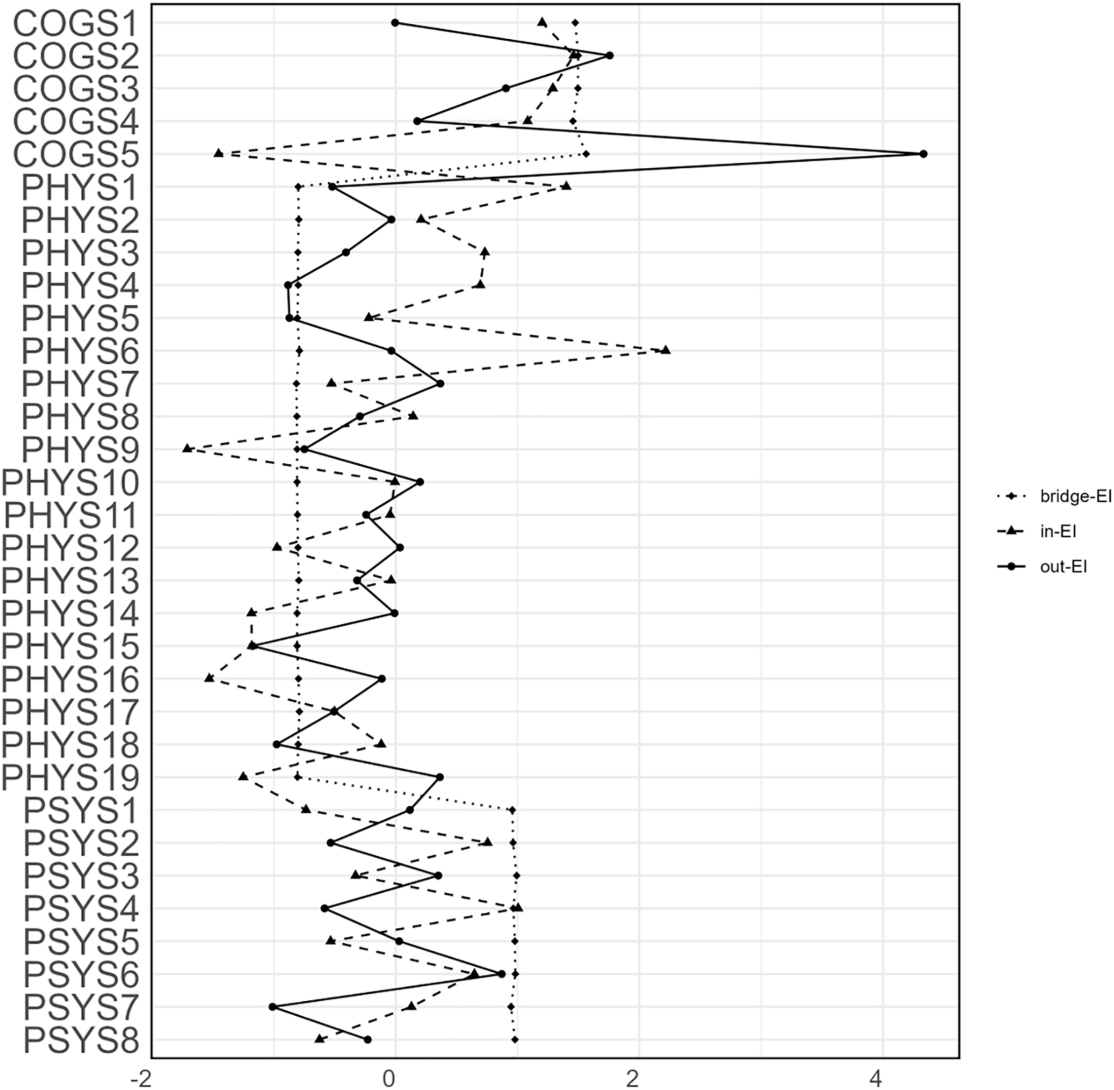
Cross-Lagged Centrality (Z-Scored). **Symptom centrality estimates in the T1 → T2 network. Larger values reflect greater centrality.** PHYS1: Fatigue; PHYS2: Dizziness; PHYS3: Headache; PHYS4: Fever; PHYS5:Cough; PHYS6: Sleep disturbance; PHYS7:Vision blur; PHYS8: Rash; PHYS9: Mouth ulcer; PHYS10: Muscle/joint ache; PHYS11: Hand/foot pain; PHYS12: Appetite loss; PHYS13: Bloating/abdominal pain/diarrhea; PHYS14: Nausea/vomit; PHYS15: Lipodystrophy; PHYS16: Weight loss; PHYS17: Low sex drive; PHYS18: Hair loss; PHYS19:Shortness of breath; COGS1: Having difficulty in concentrating; COGS2: Slow react; COGS3: Memory loss; COGS4: Having difficulty in reasoning; COGS5: Becoming confusing; PSYS1: Feeling fearful; PSYS2: Hardly focusing on anything; PSYS3: Worry overwhelmed me; PSYS4: Feeling uneasy; PSYS5:Feeling worthless; PSYS6:Feeling helpless; PSYS7: Feeling downhearted; PSYS8: Feeling hopeless.

Centrality estimates are illustrated in **Fig 4**. The symptom becoming confusing (COGS5) exhibited the highest out-EI, significantly surpassing 12 of the 31 remaining symptoms in the network (refer to **S2 Fig**). Similarly, slow react (COGS2) displayed a high out-EI, exceeding the out-EI of 30 other symptoms. In contrast, lipodystrophy (PHYS15) recorded the lowest out-EI values. According to the In-EI estimates, sleep disturbance (PHYS6) demonstrated the highest in-EI, indicating other symptoms strongly predicted it. This symptom had a significantly greater in-EI than 30 other 31 symptoms. The symptom with the lowest in-EI was mouth ulcer (PHYS9), with an in-EI considerably lower than that of 24 other symptoms, implying it was predicted to a minimal degree by the other symptoms. In addition, the symptom with the highest bridge-EI was becoming confusing (COGS5).

### Accuracy and stability of network

The confidence intervals (CIs) at a 95% level for the edge weights, which were generated using the non-parametric bootstrap technique, are illustrated in **S3 Fig**. These CIs are narrow and show considerable overlap with the original network edge weights, suggesting that the network’s edge estimates are quite precise. By utilizing the bootstrap technique, we identify a stronger correlation between the sequence of network centrality indicators derived from the bootstrap samples and those originating from the original network (see **S4 Fig**). The CS coefficient of the centrality index for the T1 → T2 network fulfills stability criteria, with out-EI, in-EI, and bridge-EI CS coefficients recorded at 0.28, 0.36, and 0.75, respectively. This indicates that the estimates for the centrality indicators out-EI, in-EI, and bridge-EI are both stable. The outcomes from the centrality difference analysis across T1 → T2 network reveal that, the symptom that demonstrates the highest centrality index (bridge-EI) exhibits a statistically significant difference when compared to most other symptoms. Conversely, the differences in out-EI and in-EI are relatively less significant.

## Discussion

This research utilized a cross-lagged panel network framework to demonstrate the distinct longitudinal and directional connections among physical, cognitive, and psychological symptoms in PWH and to determine the temporal precedence. Through our analysis, we identified bridge symptoms, central symptoms, and changes in symptom-symptom relationships within this network. These results indicated a significant level of interrelatedness among symptoms in the network, while considerable variability was noted in the prominence of individual symptoms, even within the same category. Examining these temporal relationships deepens our comprehension of symptom origins and may help pinpoint critical areas for early intervention.

This study found no significant demographic differences between participants who completed both follow-ups and those who only participated in the first, suggesting an unbiased sample of individuals lost to follow-up. However, significant immunovirological disparities were observed in the attrition group, characterized by lower CD4 counts, higher proportions of recent ART initiators, and a greater comorbidity burden. This clinical profile reflects: active viral replication driving systemic inflammation and fatigue; ART adaptation challenges during early treatment phases; and comorbidity-HIV synergy exacerbating functional impairment. Collectively, these factors constitute a pathophysiological burden that impedes consistent study engagement, consistent with the ‘burden of treatment’ paradigm in chronic disease populations [23]. Similar studies have shown that poor physical and mental health is closely linked to low treatment adherence in patients with chronic diseases. [24–26]

During the 3-month follow-up, 85 PWH were lost to follow-up, likely due to more severe disease and greater autoimmunity, which could result in more pronounced symptoms. The loss of these individuals led to a decrease in self-reported symptoms, aligning with the study’s findings. While ART duration varied, symptom trajectories in virologically suppressed PWH are typically stable beyond the initial adaptation phase (≥4–8 weeks post-ART initiation). This stability reduces concerns that ART duration confounds short-term symptom fluctuations. Empirical testing confirmed no association between ART duration and symptom change scores. Comorbidity burden was low (mean CCI 0.4) and uncorrelated with symptom severity; sensitivity analyses confirmed negligible impact on network estimates. Notably, hand/foot pain was reported more frequently at the 3-month follow-up compared to the initial report, suggesting disease progression or the onset of comorbidities in the 706 PWH who remained in the study. Additionally, symptoms such as hopelessness and weight loss showed an increasing trend in the second report, indicating a deterioration in depression and nutritional status as the disease progresses. These findings support studies by Alebel [27] and Madundo [28], which highlight the importance of dynamic symptom management. Moreover, the research by Alum [29] emphasizes the interaction between depression and malnutrition in PWH, suggesting the need for an integrated model of HIV management that includes both nutritional counseling and mental health services.

This study established China’s first cross-lag network model for symptomatology in PWH. The results show that over the three-month treatment period, the symptom of ‘becoming confusing’ is linked to the highest number of other symptoms in the entire network, followed by ‘slow reaction,’ which predicts several different symptoms. Both symptoms belong to the cognitive dimension, indicating that early cognitive symptoms have a significant impact on the emergence or worsening of other symptoms over time. Additionally, the study highlights ‘becoming confusing’ as a key bridging symptom that connects different symptom dimensions, including physical symptoms (such as ‘cough’ and ‘headache’) and psychological symptoms (such as ‘difficulty focusing’ and ‘overwhelming worry’). Bridging symptoms are central symptoms that link different symptom clusters or disease modules, facilitating the spread or association between them [21,30]. The results emphasize the amplifying role of cognitive symptoms within the overall symptom network. Once the symptom of ‘becoming confusing’ appears or worsens, it can trigger a cascade of effects, leading to both physical and psychological discomfort for patients, potentially accelerating disease progression and new symptoms to emerge. The development of comorbidities can occur significantly within a short period, such as three months. Previous studies on contemporaneous networks have identified ‘becoming confusing’ as a central symptom within the overall networks [17]. While this study incorporates a temporal dimension, the network changes and findings from contemporaneous networks remain consistent. These results not only shed light on the mechanisms of mutual association between physical, psychological, and cognitive symptoms [31] but also highlight the importance of cognitive symptoms as key targets for disease management by clinical medical staff. Recent research indicates that cognitive impairment among PWH is a growing global concern, with the prevalence of HIV-related neurocognitive disorders estimated at around 42.6% [32]. This significantly affects patients’ quality of life. As a result, improving early assessment and monitoring of cognitive symptoms, along with increasing awareness of these symptoms, is essential for effective symptom management. This approach also opens up new opportunities for transforming intervention strategies and care paradigms for healthcare professionals.

Given that participants lost to follow-up had more severe clinical profiles at baseline, it is plausible that they also experienced a greater and more complex symptom burden at the time of the second assessment. Therefore, the symptom interactions identified in our analysis, which are based on data from a potentially more resilient subpopulation that remained in care, might represent a conservative estimate of the true network dynamics. The symptom networks in the broader patient population, particularly among those with advanced disease or poorer engagement, are likely to be more densely interconnected and intense than what we observed. Consequently, our findings should be interpreted as providing valuable insights into the longitudinal symptom experiences of a specific group—patients who were clinically stable enough to be retained in the study. They may not fully capture the experiences of the most vulnerable patients who are often lost to follow-up, thus highlighting a critical area for future research aimed at capturing the full spectrum of illness experience in this population.

Although this research offers essential perspectives on the individual-level dynamic characteristics of symptoms experienced by PWH, several limitations should be acknowledged. Firstly, viewing the findings as hypotheses rather than definitive evidence of causal links is critical [33]. Secondly, the heavy reliance on self-reporting measures could lead to participant-reporting bias, mainly due to heightened cognitive demands and fatigue from numerous follow-up appointments. Thirdly, while CLPN can suggest the potential direction of links, the ideal time intervals needed to accurately capture the relationships among symptoms remain unclear. Fourthly, the directionality inferred from cross-lagged paths should be interpreted with caution, as it reflects statistical prediction rather than true causality. Our study utilized a three-month interval. Researchers should carefully consider the appropriate time lag, and sampling frequency. Structural barriers limit rural care engagement. This urban bias necessitates caution in extending findings to rural PLWH, who may experience distinct symptom profiles due to delayed diagnosis, fragmented care, and socioeconomic stressors. While socioeconomic factors were not explicitly modeled, their high temporal stability in this short-term study likely minimized confounding bias for symptom network estimation. Future longer-term studies should incorporate these variables to examine their moderating effects on symptom cascades.

## Conclusion

Dynamic changes in symptoms are inevitable. The exploration of future disease trends, mainly through dynamically changing symptom relationships, has consistently been a significant concern in clinical practice. A symptom network analysis using a cross-lagged model in this study provides a fresh perspective. Our findings indicate that cognitive symptoms, represented primarily by nodes such as having difficulty in concentrating, also show high connectivity with physical symptoms, particularly cough and sleep disturbance. Notably, mouth ulcers appear to be particularly susceptible to other symptoms. Therefore, enhancing the assessment and monitoring of cognitive status at an early stage should be a critical component of clinical nursing practice.

## Supporting information

S1 Fig Autoregressive edges for each symptom in the network. (TIF)

S2 Fig Centrality difference tests for the network with T1 predicting T2. Black boxes indicate symptoms that significantly differ in centrality (*P* < .05), and gray boxes means no significant differences among symptoms. PHYS1: Fatigue; PHYS2: Dizziness; PHYS3: Headache; PHYS4: Fever; PHYS5:Cough; PHYS6: Sleep disturbance; PHYS7:Vision blur; PHYS8: Rash; PHYS9: Mouth ulcer; PHYS10: Muscle/joint ache; PHYS11: Hand/foot pain; PHYS12: Appetite loss; PHYS13: Bloating/abdominal pain/diarrhea; PHYS14: Nausea/vomit; PHYS15: Lipodystrophy; PHYS16: Weight loss; PHYS17: Low sex drive; PHYS18: Hair loss; PHYS19:Shortness of breath; COGS1: Having difficulty in concentrating; COGS2: Slow react; COGS3: Memory loss; COGS4: Having difficulty in reasoning; COGS5: Becoming confusing; PSYS1: Feeling fearful; PSYS2: Hardly focusing on anything; PSYS3: Worry overwhelmed me; PSYS4: Feeling uneasy; PSYS5:Feeling worthless; PSYS6:Feeling helpless; PSYS7: Feeling downhearted; PSYS8: Feeling hopeless.
(TIF)

S3 Fig Bootstrapped 95% confidence intervals have been calculated for each edge weight in the networks, facilitating the prediction of T2 from T1. The red lines represent the edge weights in the estimated sample network.
(TIF)

S4 Fig Stability of centrality measures for networks using T1 to predict T2. (TIF)
