# Supplementary figures and images for: The dynamic network characteristics of physical, psychological, and cognitive symptoms of people with HIV based on cross-lagged network analysis in China
Source: PLoS One. 2026 Mar 20;21(3):e0340077. doi: 10.1371/journal.pone.0340077 (PMC13004379; doi:10.1371/journal.pone.0340077)

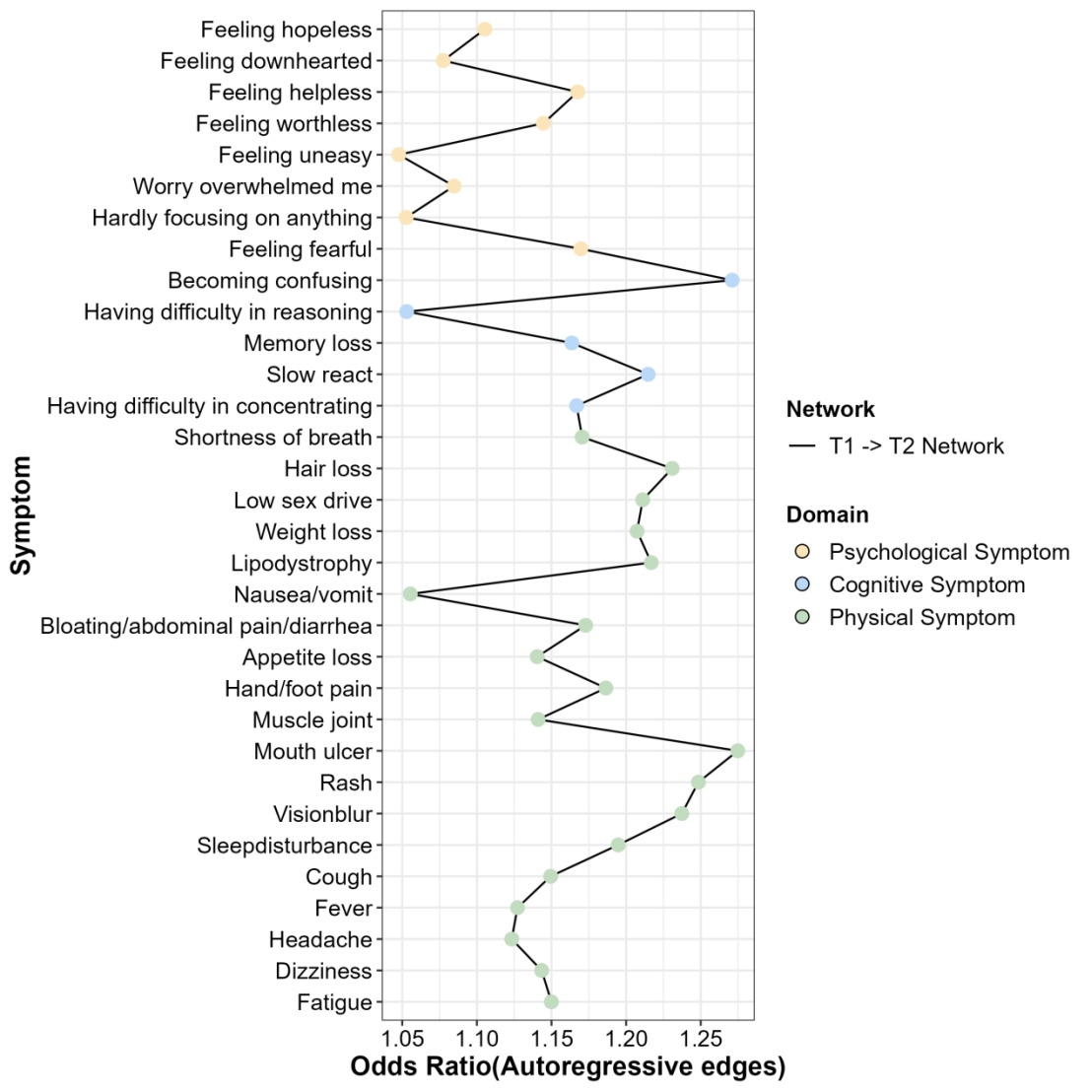

Supplement: S1 Fig — (TIF) [file pone.0340077.s001.tif]

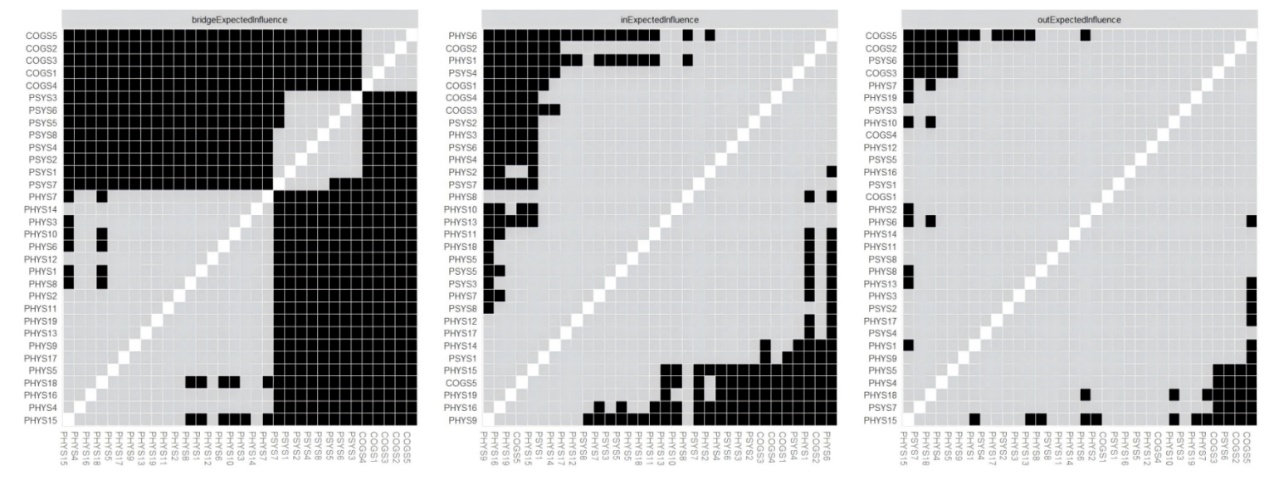

Supplement: S2 Fig — Black boxes indicate symptoms that significantly differ in centrality (P < .05), and gray boxes means no significant differences among symptoms. PHYS1: Fatigue; PHYS2: Dizziness; PHYS3: Headache; PHYS4: Fever; PHYS5:Cough; PHYS6: Sleep disturbance; PHYS7:Vision blur; PHYS8: Rash; PHYS9: Mouth ulcer; PHYS10: Muscle/joint ache; PHYS11: Hand/foot pain; PHYS12: Appetite loss; PHYS13: Bloating/abdominal pain/diarrhea; PHYS14: Nausea/vomit; PHYS15: Lipodystrophy; PHYS16: Weight loss; PHYS17: Low sex drive; PHYS18: Hair loss; PHYS19:Shortness of breath; COGS1: Having difficulty in concentrating; COGS2: Slow react; COGS3: Memory loss; COGS4: Having difficulty in reasoning; COGS5: Becoming confusing; PSYS1: Feeling fearful; PSYS2: Hardly focusing on anything; PSYS3: Worry overwhelmed me; PSYS4: Feeling uneasy; PSYS5:Feeling worthless; PSYS6:Feeling helpless; PSYS7: Feeling downhearted; PSYS8: Feeling hopeless. (TIF) [file pone.0340077.s002.tif]

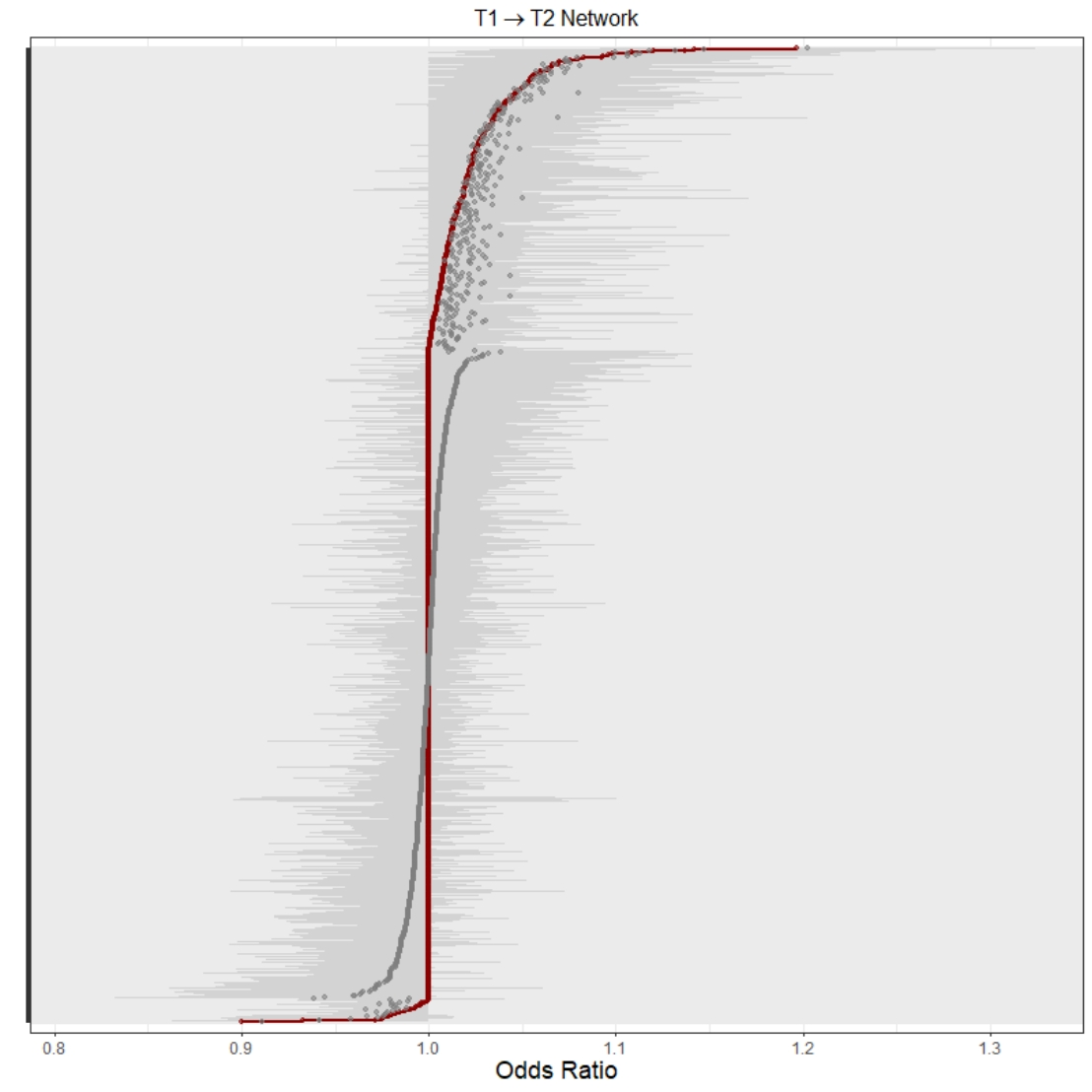

Supplement: S3 Fig — The red lines represent the edge weights in the estimated sample network. (TIF) [file pone.0340077.s003.tif]

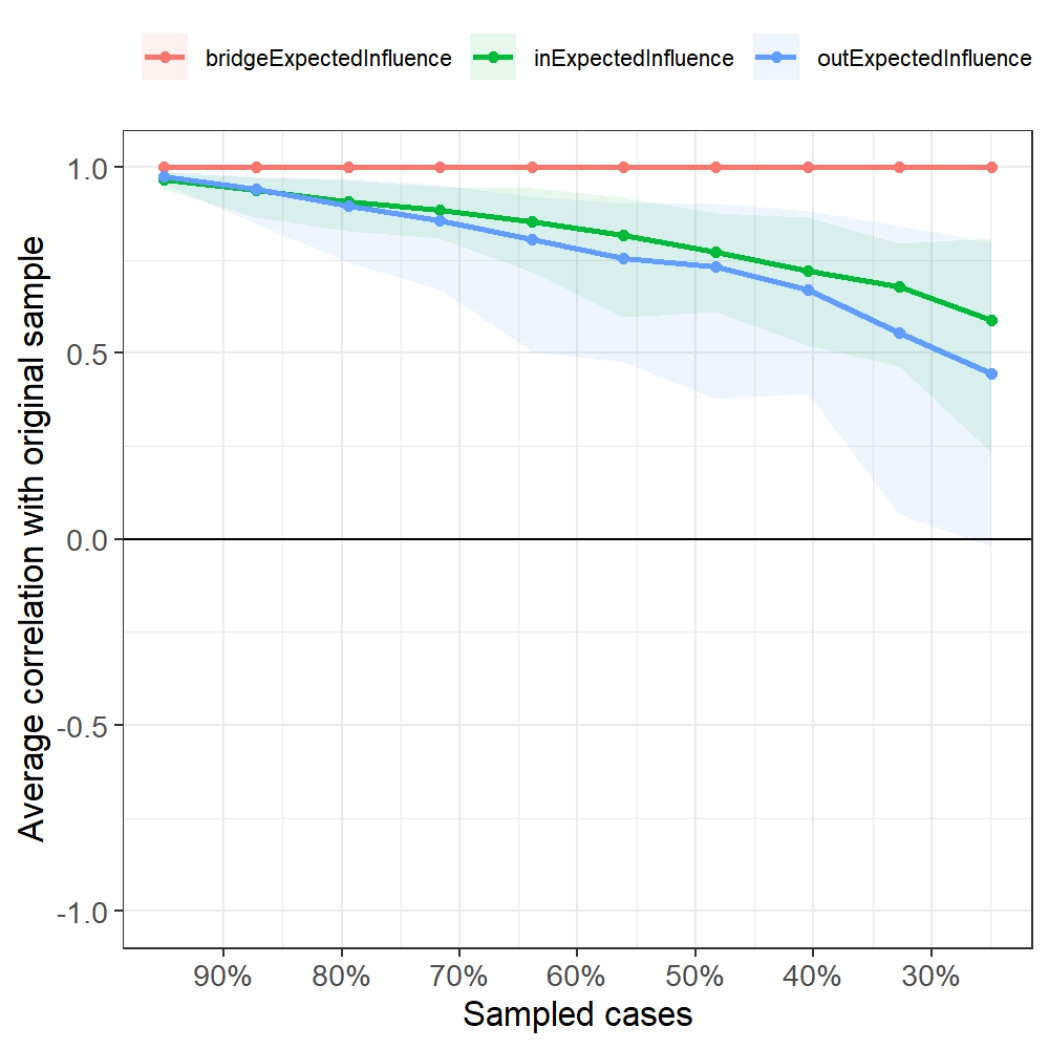

Supplement: S4 Fig — (TIF) [file pone.0340077.s004.tif]
